# Burnout among postgraduate medical trainees in Lebanon: Potential strategies to promote wellbeing

**DOI:** 10.3389/fpubh.2022.1045300

**Published:** 2023-01-06

**Authors:** Aline Yacoubian, Jad A. Degheili, Asdghig Der-Boghossian, Jad Najdi, Rebecca Andraos, Salah Zeineldine

**Affiliations:** ^1^Department of Surgery, American University of Beirut Medical Center, Beirut, Lebanon; ^2^Department of Urology, Children's Hospital of Eastern Ontario (CHEO), Ottawa, ON, Canada; ^3^Department of Internal Medicine, American University of Beirut Medical Center, Beirut, Lebanon

**Keywords:** postgraduate medical trainees, COVID-19, burnout—professional, wellbeing, leadership and physicians, program directors

## Abstract

**Objective:**

Burnout is a widespread issue in healthcare for many years. Lebanon combatted political and economic crises before the coronavirus disease 2019 (COVID-19) pandemic, in addition to the port explosion in August 2020. The study aimed to identify the determinants of personal burnout, patient-related burnout, and work-related burnout among postgraduate medical trainees (PGMT) and evaluate its relationship with sociodemographic characteristics.

**Design:**

A cross-sectional study utilized the Copenhagen Burnout Inventory (CBI) involving electronic, voluntary, and anonymous survey. The survey was completed by 188 PGMT including residents and fellows from all specialties and all levels of training.

**Results:**

The prevalence rates are 68.6% for personal burnout, 63.3% for work-related burnout, and 35.1% for patient-related burnout.

**Conclusion:**

Results improve our understanding of the phenomenon of burnout, and the role of program leadership in shaping the impact of burnout on training and promoting wellbeing of PGMT. Discussion focuses on providing potential wellbeing strategies for program directors to follow for mitigating burnout.

## Introduction

Burnout is a complicated concern in the occupational sector that does not have a standard definition (1). The World Health Organization (WHO) reclassified workplace burnout in the 11th Revision of the International Classification of Diseases (ICD-11). Burnout depicts the following symptoms: Feelings of energy depletion or exhaustion; increased mental distance from one's job, or feelings of negativism or cynicism related to one's job and reduced professional efficacy (2). Herbert Freudenberger first described burnout in the 1970's and referred to it as worsening, wearing out, or becoming tired by making extreme demands on energy, strength, or resources (3). It regularly happens ~1 year after someone has begun working in an organization, when several elements come into play (3). Burnout manifests as a steady emotional depletion and loss of motivation and the ones who are prone to it are those who are dedicated and committed individuals (3). It can lead to physical and psychological problems, and compromised mental health (4). It is a contagious concept wherein those who experience burnout can negatively influence their colleagues by personal conflict and disruption of work assignments (1). Burnout was initially described as a syndrome for those who work with people, i.e., “human services” (5) prior to including information and other services (6). Burnout is widely illustrated as a syndrome of emotional exhaustion, depersonalization, and reduced personal accomplishment (7).

Burnout is common in medicine due to the demanding and challenging profession (8). It arises in graduate medical training wherein the prevalence has ranged between 45 and 71% in medical students (8) and in a recent study among 315 medical students in China, the prevalence rate was 45.9% (9). Burnout continues throughout postgraduate medical training and physicians' career (10). There has been a surge in mental illness and suicides in the medical community in the past years (11). Extensive research has been performed in the field of healthcare to alleviate burnout (1). Postgraduate medical trainees (PGMT) represent a unique occupational group with an increased risk of burnout. Their wellbeing is an important organizational asset since it is linked to performance and safety behavior (12). Recently, the coronavirus disease 2019 (COVID-19) pandemic had a dramatic strain on the overall residency and fellowship training especially on the clinical and procedural workload and educational activities (13, 14). For example, PGMT in charge of patients with COVID-19 are more prone to feel anxious and stressed (15).

Several factors lead to burnout during the pandemic, and they comprise: elongated duty hours, longer patients' list, sicker patients, further responsibility, fear of getting infected and infecting colleagues, friends, and family members (16). PGMT who directly care for infected patients or are redeployed to COVID-19 units, are most likely to feel so too because of increased exposure to the virus which is associated with fear (17). Other factors include shortages of personal protective equipment, daily changes in shifts, further work, obligations to family and others outside the hospital premises, and provision of childcare (15).

Lebanon has plunged into one of the most cataclysmic times in its history with economic, financial, and social collapse. Challenges that arose include sudden drop of the national currency over 80%, inflation of prices by 200% and deterioration of the banking sector and brain drain of skilled employees (18). The Beirut Port explosion in August 2020 that resulted in about 220 deaths ruined the city and caused massive infrastructure damage and homelessness. Other challenges include partial removal of subsidies on essential medicines, diesel and other basic needs, severe shortages of medicine and increase of price of medications and cost of treatment for chronic diseases. Diesel prices rose around 2,000 per cent within a year, which made it very difficult and costly for most workers to report to work (18). The wiping out of lifetime savings by the banking sector, wherein the politically well-connected and wealthy individuals transferred their capital out of Lebanon made a substantial impact on the economy. Nonetheless, the country had already suffered from civil wars, increased influx of Syrian and Palestinian refugees, camps, and dramatic rise in unemployment and poverty (18).

The Medical Center is a tertiary medical center located in Beirut, Lebanon. The center provides 20 residency training programs. PGMT are trained by a clinical teaching team, and each program is directed by a program director with the support of a program coordinator and the Graduate Medical Education office. The programs are accredited by the Accreditation Council for Graduate Medical Education—International (ACGME-I). The years of training are from Post Graduate Year (PGY) 1 until PGY5 (the digits representing the year of training, PGY1 = 1st year) for some programs and PGY6 for other sub-specialties. The 7th year (PGY7) is for fellows. A previous study conducted in two academic hospitals in Beirut, Lebanon reported a worrying rate of burnout among Lebanese medical residents across both public and private hospitals (19) amidst the difficult and challenging circumstances that the country has been struggling with for decades especially the war in 2006 and the assassinations of politicians and regular bombings in random regions. All these factors have a great toll on the mental health of citizens in general. The primary objective of the study is to evaluate the sociodemographic characteristics of PGMT and their relationship with the different scales of burnout given by a validated instrument.

## Methods

### Study design and sample

#### Instrument

An anonymous web-based questionnaire was conducted to assess burnout using the well-known inventory called the Copenhagen Burnout Inventory (CBI). The core of burnout in this tool is fatigue and exhaustion which is in conformity with the historical development of the theory of burnout, and with recent definition by leading researchers in the field which have emphasized these keywords (20). The author explained that there is a feature in addition to fatigue and exhaustion that needs to be addressed in the instrument. This feature is the attribution of fatigue and exhaustion to domains in an individual's life which is work and specifically client work (20) and for these reasons the author created the CBI.

The CBI is a validated tool and consists of three sub-dimensions: personal burnout, work-related burnout, and client-related burnout (20). The three scales are intended to be applied in different domains. The personal burnout questions represent an actual generic scale that anyone can answer and a general state of fatigue and exhaustion. The work-related burnout questions are for people with paid work and represents the degree of burnout perceived by the person to be related to his/her work. This scale can only be used if people have work of some kind. The client-related burnout questions include the term “client” (or a similar term when appropriate such as patient, student, etc.). This is the level of burnout perceived by the person as related to working with clients (patients, students, children, etc.). The use of this scale is restricted to employees who work with clients (20). The client-related burnout will be replaced by patient-related burnout in this study. The three subscales have 19 questions in total distributed as follows: The personal burnout has six questions; the work-related burnout has seven questions and the client-related burnout has six questions. Ratings are given based on a five-point Likert scale. Each item is scored from 0 to 100 (0 = never/almost never, 25 = seldom, 50 = sometimes, 75 = often, 100 = always) (20). Although the author of the CBI does not recommend the use of cutoff scores and dichotomization, burnout was defined as high and low burnout, in several studies, using the cutoff score of 50 that is the middle value of 0–100-point for each scale (21).

### Data collection

The target population consisted of PGMT of all levels of training including residency and fellowship programs. The electronic survey was anonymous and confidential and was distributed electronically. An email was sent to all PGMT on February 15, 2022, through the Lime Survey portal. There were three reminders sent exactly 1 week apart from the initial email. PGMT were included if they were male and female residents or fellows, aged 18 years and above, employed by the institution and consented to participate in the survey. The survey was voluntary and did not include any personal identifiers. The Institutional Review Board (IRB) of the university approved the study (SBS-2021-0219). The email presented the name of the principal investigator and study coordinator and the objectives of the study. Those who were interested to participate had to click on the link provided in the email. The documentation of written consent was waived to avoid collection of identifying information and due to the online nature of the survey. Participants were required to verify that they consented to participate before beginning the survey. They were informed of the anonymity of the study and the protection rights of the data. The questionnaire included the 19 original questions from the three dimensions found in the CBI and at the end included demographic questions such as gender, age, marital status, and year of training. We decided to use the three scales from the CBI to capture all the different dimensions of burnout for a better understanding of the theory. Only valid participants were included in the analysis. Missing questionnaires were excluded.

### Statistical analysis

In the descriptive analysis of the sample, summary statistics were applied as appropriate. The mean values were calculated for the subscales of the CBI. The Independent *T*-test was used for comparison of mean scores in two groups and if there were more than two groups involved, we used the one-way analysis of variance (ANOVA). Reliability of the test was assessed by measuring Cronbach's alpha. For descriptive analysis, variables were presented with mean, and standard deviation (SD) for continuous data and with frequencies and percentages for categorical data. The mean item score was calculated per scale based on the proposed scoring system of the questionnaire (20). The Independent *T*-test was used to compare means between two groups, whereas the ANOVA to compare between three groups or more, after checking for homogeneity of variances. Binomial Logistic Regression was used to explore association extent between the demographic variables and the three scales: personal, work-related, and patient-related burnout.

Cronbach's alpha was determined to measure the internal consistency or reliability of the survey items. The whole inventory had a score of (α = 0.938) and each one separately as follows: personal burnout (α = 0.895), work-related burnout (α = 0.900), and patient-related burnout (α = 0.912). The collected data was entered into Microsoft Excel with a double-entry method to avoid errors. Statistical Package for the Social Sciences (SPSS; IBM Corp. Released 2020. IBM SPSS Statistics for Windows, Version 27.0. Armonk, NY: IBM Corp) was used. Demographic variables such as gender, marital status and year of training were included as independent variables. Age was not used in our analysis since the range was very close in the sample (26–32 years) and a previous study conducted in Lebanon showed that age was not related to burnout (19). A *p*-value of < 0.05 was considered statistically significant.

## Results

Survey invitations were sent to 389 PGMT. There were 213 unique responses collected. There were 25 missing items either in the questions or in the demographic information and hence were excluded. The final sample comprised 188 valid responses with a response rate of 48.32%.

Descriptive statistics are presented in Table 1. Most participants are females (60.1%). The range of age is 26–32 years for both males and females. Approximately half of the participants are single (55.3%), others in a relationship (26.6%), while 17.0% are married and 1.1% are divorced. Most trainees are in the 1st year of training (27.7%), followed by 3rd year (22.3%), 4th year (18.6%), 2nd year (13.8%), 5th year (9.0%), 6th year (7.4%), and 7th year (1.1%).

**Table 1:** Demographic characteristics of the trainees (*n* = 188).

**Gender**	**Frequency (%)**
Females	113 (60.1)
Males	75 (39.9)
**Marital status**	
Single	104 (55.3)
In a relationship	50 (26.6)
Married	32 (17.0)
Divorced/separated	2 (1.1)
Year of training^*^	
PGY1	52 (27.7)
PGY2	26 (13.8)
PGY3	42 (22.3)
PGY4	35 (18.6)
PGY5	17 (9.0)
PGY6	14 (7.4)
PGY7	2 (1.1)

* The years of training are from Post Graduate Year (PGY) 1 until PGY5 (the digits representing the year of training, PGY1 = 1st year) for some programs and PGY6 for other sub-specialties. The 7th year (PGY7) is for fellows.

Tables 2A–C shows the detailed scales presented in frequencies, percentages, mean and SD scores. Among its subscales, personal burnout was highest (mean = 59.17), followed by work-related burnout (mean = 54.86) and client-related burnout (mean = 37.69). The highest mean score for items in each of the dimensions were as follows: “How often do you feel tired?” (mean = 71.14), followed by “How often are you emotionally exhausted” (mean = 65.69), “How often are you physically exhausted?” (mean = 65.42) for personal burnout. For work-related burnout, “Do you feel worn out at the end of the working day?” (mean = 65.82), “Is your work emotionally exhausting?” (mean = 62.76), and “Do you feel burnt out because of your work?” (mean = 57.57). For patient-related burnout, “Do you feel that you give more than you get back when you work with patients?” (mean = 53.85), “Does it drain your energy to work with patients?” (mean = 38.43), and “Do you find it hard to work with patients?” (mean = 35.10). The prevalence rate is as follows: 68.6% for personal burnout, 63.3% for work-related burnout, and 35.1% for patient-related burnout.

**Table 2A:** Frequency distribution, mean and SD of the personal burnout scale.

	**Personal burnout**					
	**Frequency (%)**					**Mean (SD)**
	**Never/ almost never (0)**	**Seldom (25)**	**Sometimes (50)**	**Often (75)**	**Always (100)**	
How often do you feel tired?	-	2 (1.1)	56 (29.8)	99 (52.7)	31 (16.5)	71.14 (17.39)
How often are you physically exhausted?	3 (1.6)	13 (6.9)	63 (33.5)	83 (44.1)	26 (13.8)	65.42 (21.66)
How often are you emotionally exhausted?	3 (1.6)	16 (8.5)	64 (34.0)	70 (37.2)	35 (18.6)	65.69 (23.40)
How often do you think: “I can't take it anymore?”	21 (11.2)	49 (26.1)	60 (31.9)	47 (25.0)	11 (5.9)	47.07 (27.20)
How often do you feel worn out?	4 (2.1)	31 (16.5)	64 (34.0)	72 (38.3)	17 (9.0)	58.90 (23.34)
How often do you feel weak and susceptible to illness?	22 (11.7)	50 (26.6)	64 (34.0)	34 (18.1)	18 (9.6)	46.80 (28.33)
Total		59.17 (19.29)

**Table 2B:** Frequency distribution, mean and SD of the work-related burnout scale.

	**Work-related burnout**					
	**Frequency (%)**					**Mean (SD)**
	**Never/ almost never**	**Seldom**	**Sometimes**	**Often**	**Always**	
Do you feel worn out at the end of the working day?	-	20 (10.6)	67 (35.6)	63 (33.5)	38 (20.2)	65.82 (23.10)
Are you exhausted in the morning at the thought of another day at work?	11 (5.9)	37 (19.7)	60 (31.9)	50 (26.6)	30 (16.0)	56.78 (28.15)
Do you feel that every working hour is tiring for you?	24 (12.8)	60 (31.9)	61 (32.4)	30 (16.0)	13 (6.6)	43.05 (27.33)
Do you have enough energy for family and friends during leisure time? (reversed scoring)	10 (5.3)	48 (25.5)	73 (38.8)	44 (23.4)	13 (6.9)	50.22 (24.79)
Is your work emotionally exhausting?	8 (4.3)	20 (10.6)	62 (34.0)	64 (34.0)	34 (18.1)	62.76 (26.04)
Does your work frustrate you?	24 (12.8)	38 (20.2)	72 (38.3)	39 (20.7)	15 (8.0)	47.73 (27.81)
Do you feel burnt out because of your work?	15 (8.0)	22 (11.7)	69 (36.7)	55 (29.3)	27 (14.4)	57.57 (27.58)
Total		54.86 (17.49)

**Table 2C:** Frequency distribution, mean and SD of the patient-related burnout scale.

	**Patient-related burnout**					
	**Frequency (%)**					**Mean (SD)**
	**Never/ almost never (0)**	**Seldom (25)**	**Sometimes (50)**	**Often (75)**	**Always (100)**	
Do you find it hard to work with patients?	41 (21.8)	58 (30.9)	66 (35.1)	18 (9.6)	5 (2.7)	35.10 (25.41)
Does it drain your energy to work with patients?	33 (17.6)	59 (31.4)	34 (34.0)	26 (13.8)	6 (3.2)	38.43 (25.90)
Do you find it frustrating to work with patients?	44 (23.4)	62 (33.0)	56 (29.8)	22 (11.7)	4 (2.1)	34.04 (25.81)
Do you feel that you give more than you get back when you work with patients?	24 (12.8)	29 (15.4)	62 (33.0)	40 (21.3)	33 (17.6)	53.85 (31.28)
Are you tired of working with patients?	51 (27.1)	64 (34.0)	55 (29.3)	11 (5.9)	7 (3.7)	31.25 (25.93)
Do you sometimes wonder how long you will be able to continue working with patients?	57 (30.3)	50 (26.6)	54 (28.7)	14 (7.4)	13 (6.9)	33.51 (29.60)
Total		37.69 (22.86)

The Independent *T*-test in Table 3 shows that there is a statistical difference between males and females in experiencing personal burnout (*p* = 0.002) with the mean of female PGMT is higher than that of male PGMT (62.68 vs. 53.88). The mean of work-related burnout (*p* = 0.067) and patient-related burnout (*p* = 0.408) is almost the same for females and males. For marital status, the One-Way ANOVA test shows that the difference between the means of personal burnout is statistically significant (*p* = 0.033) with the highest mean for those who are single vs. those who are divorced (60.97 vs. 25.00; Table 4A). There is a statistical difference between the means of work-related burnout for the different marital status groups (*p* = 0.008) with the highest mean for those who are single vs. those who are divorced (56.25 vs. 19.64). The One-Way ANOVA test reports that the means of personal burnout are statistically significant for the different years of training (*p* = 0.010) with the highest mean for those who are PGY1 vs. those who are PGY7 (62.98 vs. 27.08; Table 4B). The result is similar for work-related burnout (*p* = 0.014) with the highest mean for those who are PGY1 vs. those who are PGY7 (57.00 vs. 32.14). The mean of patient-related burnout is almost the same across all groups in years of training.

**Table 3:** Independent *T*-test between the mean of the three scales and gender.

	**Gender**	**Mean**	**SD**	***P*-value**
Personal burnout	Females	62.68	18.23	0.002
	Males	53.88	19.75	
Work-related burnout	Females	56.76	17.36	0.067
	Males	52.00	17.41	
Patient-related burnout	Females	38.82	22.04	0.408
	Males	36.00	24.08	

**Table 4A:** One Way ANOVA Test between the mean of the three scales and marital status.

	**Marital status**	**Mean**	**SD**	***P*-value**
Personal burnout	Single	60.97	19.26	0.033
	In a relationship	59.41	16.83	
	Married	55.07	21.34	
	Divorced	25.00	11.78	
Work-related burnout	Single	56.25	17.16	0.008
	In a relationship	56.42	16.05	
	Married	50.11	18.55	
	Divorced	19.64	7.57	
Patient-related burnout	Single	38.78	23.11	0.340
	In a relationship	39.16	21.12	
	Married	33.20	24.60	
	Divorced	16.66	22.86	

**Table 4B:** One Way ANOVA Test between the mean of the three scales and year of training.

	**Year of training**	**Mean**	**SD**	***P*-value**
Personal burnout	PGY1	62.98	16.92	0.010
	PGY2	61.53	23.13	
	PGY3	63.49	17.95	
	PGY4	54.16	19.28	
	PGY5	55.14	12.45	
	PGY6	49.70	23.02	
	PGY7	27.083	14.73	
Work-related burnout	PGY1	57.00	15.54	0.014
	PGY2	59.47	22.40	
	PGY3	58.50	15.29	
	PGY4	51.12	14.94	
	PGY5	51.89	16.56	
	PGY6	43.62	20.73	
	PGY7	32.14	20.20	
Patient-related burnout	PGY1	40.38	22.43	0.110
	PGY2	44.55	23.94	
	PGY3	39.98	22.95	
	PGY4	34.88	22.80	
	PGY5	29.16	25.34	
	PGY6	28.57	13.85	
	PGY7	16.66	17.67	

Binary Logistic Regression analysis was conducted to define significant predictors of personal, work-related, and patient-related burnout (Table 5). The variables used were gender, marital status, and year of training. For analysis purposes, those who are in a relationship and those who are married are grouped in one new cluster. Those who are single and divorced are grouped in one cluster and used as the reference group. Those who are in the 6th year of training and 7th year of training are grouped together. The reason is that the number of those who are separated/divorced and the number of those in the 7th year of training is small and to yield better results. Gender is a significant predictor of personal burnout (*p* = 0.001). Males have 30.8% less risk of experiencing personal burnout than females (*p* = 0.001, 95% CI = 0.154–0.613). Trainees in the 6 and 7th year have 13.1% less risk of personal burnout (*p* = 0.002, 95% CI = 0.036–0.480), 11.3% less risk of work-related burnout (*p* = 0.002, 95% CI = 0.028–0.454), and 20% less risk of patient-related burnout (*p* = 0.047, 95% CI = 0.041–0.975) than those in the 1st year of training.

**Table 5:** Binary Logistic Regression for the scales and gender, marital status, and year of training.

**Personal burnout**			
	**Odds ratio**	**CI**	***P*** **-value**
Gender	0.308	0.154–0.613	0.001
Reference (Female)			
Marital status	1.771	0.848–3.697	0.128
Reference (Single and Divorced)			
Year of training
Reference (PGY1)
PGY2	0.916	0.302–2.773	0.876
PGY3	1.293	0.462–3.623	0.624
PGY4	0.419	0.152–1.153	0.092
PGY5	0.416	0.121–1.435	0.165
PGY6 and PGY7	0.131	0.036–0.480	0.002
**Work-related burnout**
Gender	0.744	0.391–1.417	0.368
Reference (Female)			
Marital status	1.091	0.559–2.132	0.789
Year of training
Reference (PGY1)
PGY2	1.104	0.399–3.052	0.849
PGY3	1.345	0.544–3.326	0.521
PGY4	0.697	0.277–1.756	0.444
PGY5	0.895	0.273–2.938	0.855
PGY6 and PGY7	0.113	0.028–0.454	0.002
**Patient-related burnout**
Gender	0.952	0.502–1.805	0.881
Reference (Female)			
Marital status	0.911	0.472–1.758	0.781
Reference (Single and Divorced)			
Year of training
Reference (PGY1)
PGY2	0.724	0.272–1.926	0.518
PGY3	1.137	0.499–2.592	0.760
PGY4	0.562	0.218–1.444	0.231
PGY5	0.439	0.122–1.575	0.207
PGY6 and PGY7	0.200	0.041–0.975	0.047

## Discussion

The study took place in a tertiary medical center in Beirut, which is the capital of Lebanon, and this center has the largest number of PGMT in various specialties of medicine. The author of the CBI has stated in case any scholar would like to report an overall scale for burnout from the CBI, he/she should use the scale of the personal burnout. Our study showed that the mean of 188 PGMT for personal burnout is 59.17% and the prevalence rate of personal burnout is 68.6%. The following studies used the same inventory: In one study among 113 PGMT in Pakistan, personal burnout was the highest (mean = 49.74), followed by work-related burnout (mean = 46.99) and client-related burnout (mean = 46.13) (22). Another one among 210 PGMT in India reported 51.8% for personal burnout, 37.2% for work-related burnout (measured among 151 PGMT), and 22.47% for client-related burnout (measured among 91 PGMT) (21). Another study among 245 PGMT in Colombo, Sri Lanka showed that the prevalence of personal burnout was 41.6%, for work-related burnout 30.6% and for client-related burnout 8.9% (23).

Studies that used other inventories like the Maslach Burnout Inventory and the Oldenburg Burnout Inventory had various results (9, 11, 19, 24–27). The prevalence rate of burnout was 46.3% among 393 trainees in the United States (US) (24), 35.8% among 560 trainees in the US (25), 48.6% among 3,071 residents in Brazil (11), 33% among 427 neurosurgery residents in the US (28), and 37.4% among 340 pediatric residents in France (27). Different scores for burnout imply different explanations. The difference in the prevalence rate of burnout in studies may be partly explained by the difference of the instrument employed (25). Specialty of training plays a role in burnout. For example, pediatric trainees are less likely to be confronted with emergencies than other trainees (27) and may hence report less burnout.

The high prevalence rates seen in our study reflect the challenging conditions in the country in addition to the pandemic as explained above. All these factors have placed an unprecedented strain on the medical education, training, and wellbeing of PGMT. The most important justification for the high rate of burnout in Lebanon includes the gravities and pressures that arise for PGMT residing in Lebanon who have witnessed and experienced extremely harsh conditions engraved in uncertainty and ambiguity adding to their already stressful life. The numerous and complex factors in the country have contributed to drastic and extensive changes to their sense of value, autonomy, and mental health. This is also explained by the high mean scores for the questions in our sample that revolve around feeling tired, feeling emotionally and physically exhausted, feeling worn out and having their energy drained.

The study showed high rates of personal and work-related burnout and lower rate of patient-related burnout like other studies (23). The reason for low patient-related burnout may be that PGMT become adapted to high patient loads from the beginning of their training and therefore may not perceive patient work as a burden especially in the senior years (23). This implies that patient care is not the primary cause of burnout, but personal and work-related factors are. PGMT may adapt to patient care and load due to experience and years of training. PGMT know very well prior to training that most of their work revolves around patients and hence they become prepared for patient care. An earlier study conducted in two hospitals in Lebanon reported that the number of calls (more than eight calls per month), working continuously for more than 80 h per week, type of specialty, postgraduate years, and number of continuous working hours were statistically associated with high burnout (19). A study conducted on 387 community pharmacists in Lebanon showed that the prevalence of personal, work-related, and client-related burnout was 77.8, 76.8, and 89.7%, respectively (29).

Moreover, female trainees report higher personal burnout than male trainees and the result is statistically significant. This is like other studies (23, 29, 30) and can be explained by the variations in gender roles and cultural differences (23). Female trainees especially married ones may assume more responsibilities when it comes to work and family balance, emotional demands, family concerns and childcare for those who have children. Although marital status is not a determinant of personal burnout, we assume that female trainees may report more personal burnout because of fatigue and exhaustion emerged from their additional responsibilities due to work-life balance eventually increasing vulnerability and stress. A trainee who was a female, who worked more than 30 h and who experienced a major stress in the past 6 months had significantly increased odds of high emotional exhaustion burnout by 2.54, 2.91, and 3.38, respectively (19). Literature shows that female PGMT in Lebanon face other social pressures and burden ranging from stigma of living alone, away from family to the urgent need of relatively early marriage vs. the academic and long-year commitment of the medical profession (19). Trainees in the 6 and 7th training year reported lower risk of personal and work-related burnout vs. the 1st year of training. This is in contrast with other studies (19, 30). For example, in a study conducted in Bahrain, chief residents had higher levels of personal and work-related burnout in comparison to other job positions (30).

The wellbeing of PGMT during and after the pandemic remains a chief responsibility of leaders and program directors in graduate medical education (17). The COVID-19 pandemic presents an opportunity to reflect on assessment and support of PGMT wellbeing (17). Literature shows that employees tolerate workload if they feel that they are well-rewarded for their efforts (1). Therefore, an intervention could emphasize the areas of appreciation, value, and reward (1). Another study showed that feeling valued by leaders and program directors is associated with 66% lower odds of psychiatric symptoms and 59% lower odds of burnout (25). Another study showed that social support, particularly subjective support might offer a protecting effect against stress and hence lessen the likelihood of having burnout (9). Therefore, PGMT burnout can be overturned with leadership that places appreciation, transparency, and practical initiatives at the core of its actions.

### Theoretical and practical implications

This research makes several theoretical contributions. Our work contributes to helping literature, as previous attempts to alleviate burnout among PGMT were not very fruitful especially in lieu of the pre-existing burnout among PGMT before the COVID-19 pandemic. The narrowness of the topic of wellbeing in curbing burnout has led scholars to call for research that examines the many different roles that burnout plays in organizational settings. We shed light on when and why PGMT training should rise again to normal levels following a preceding failure by seeing the role of employee self-reflection about addressing failure to generate pragmatic solutions. PGMT should notice that their organization aligns the strategy and plan with the values of their profession wherein their voice and feedback are appreciated and acknowledged (31).

The study results show that external factors play a major role in organizational performance. The negative conditions of a certain country act as a barrier to the overall function of an institution such as a healthcare system eventually affecting the training program. While the whole world has been turned upside down with the COVID-19 pandemic, PGMT in Lebanon have additional economic and political challenges that further exacerbated their situation and quality of life. The situation in the country plays a detrimental role in the mental health of healthcare workers especially PGMT who are in training. The experience at the center shows that PGMT are surviving well-evidenced by graduating on time and securing positions in reputable centers in North America, Europe, and Gulf countries. This is aided by the continuous support from program directors, program coordinators, Graduate Medical Education office and leaders in all departments.

The authors present the following recommendations based on previous studies for leaders and program directors in graduate medical education to consider (8, 25):

- Promoting frank communication and awareness on wellbeing, burnout, and resilience.
- Demonstrating appreciation of trainees through town halls, direct messaging, and acts of gratefulness.
- Normalizing mental health and removing the stigma associated with its utilization.
- Integrating self-care and healthy lifestyle to the teaching curricula of postgraduate medical education.
- Encouraging adequate amount of sleep, physical exercise, healthy diet, and healthcare.
- Creating flexible schedules to avoid extra duty hours and superfluous administrative assignments.
- Incorporating open door policy for program directors by ensuring confidentiality and privacy.
- Increasing the number of hospitalists or part-timers if funding is available.
- Increasing time for trainees to spend with family/partners/children.
- Adapting social support through family and friends.
- Providing support programs for spouses, children, and dependents.
- Establishing wellness programs and supportive working environments with colleagues.
- Meeting to ensure that trainees meet the minimum case requirements prior to graduation. Discussing the possibility of future electives in other centers for those who need to meet the number.
- Discussing ways to compensate for missed educational opportunities and reduced job opportunities.
- Providing financial support for research and academic conferences at different levels if possible.
- Providing women especially those who are married or have children with creative avenues to stay engaged with the workplace and with their personal life.

### Limitations and directions for future research

The present study has several limitations. Burnout can be defined in numerous ways and can be assessed by different inventories. For example, numerous studies used the Maslach Burnout Inventory (27). However, the Maslach Burnout Inventory is commercially available, while the CBI is available for non-commercial research. The study was conducted in a single hospital in Beirut. This limits the ability to generalize to other institutions with postgraduate medical training program across the country. The cross-sectional design does not evaluate burnout over time, i.e., only correlation can be assessed and not causation and therefore, we cannot deduce causality between the variables. There is lack of periodic comparison of results such as pre-pandemic, intra-pandemic and post-pandemic findings which could be helpful but is challenging to obtain. Selection bias, wherein trainees with more free time may have responded to the survey, may cause under-reporting of burnout due to the difficulty of capturing residents with less spare time. Response bias in which those with burnout are more or less likely to respond to the survey. Reporter bias exists, because participants may answer how they believe others would like them to respond. PGMT may feel averted or hesitant to complete surveys due to the challenging conditions in the country, or perhaps due to doubt about the effectiveness of surveys in general. We cannot find out whether participants answered the survey questions honestly. We can only assume that they did so based on the country conditions.

Future research should assess the impact of wellbeing interventions during and post COVID-19 pandemic and the outcome on burnout. Future studies should focus on evaluating burnout among different specialties and emphasize the impact of wellness initiatives and the effect on wellbeing. It is important to pay attention to the following points: screening for burnout, providing comfort for PGMT to feel free to ask for support from their supervisors, organizing workload, improving scheduling, and working conditions, and promoting psychological wellbeing. Program directors and leaders should strive to improve resident operative autonomy by fostering positive interpersonal relationships, professionalism, conference presentations, problem solving and operative planning (32). Research questions to focus on include if burnout is a contributing factor in the migration of PGMT, what altering working conditions decrease burnout among PGMT and how to move to a smooth post-COVID-19 transition period.

## Conclusion

The COVID-19 pandemic has put a considerable toll on the physical, social, economic, and mental wellbeing of PGMT. Understanding the different factors affecting burnout is crucial to curb its occurrence. The wellbeing of PGMT is vital and should be closely followed-up even after the pandemic. Preventive strategies and potential interventions such as improved work-life balance, increased organizational and peer support, can help curtail the level of chronic stress, promote work satisfaction, improve education and training and foster career advancement and satisfaction. One of the most suitable and promising directions for theories of emergence is the opportunity to discover how and why burnout symptoms emerge with a focus on differences in demographic characteristics. Insights from these findings can guide both future research and practical interventions designed to enhance wellbeing seeking behaviors in organizations.

## Data availability statement

The original contributions presented in the study are included in the article/supplementary material, further inquiries can be directed to the corresponding author.

## Ethics statement

This study was approved by the Institutional Review Board (IRB) of the university approved the study (SBS-2021-0219). Written informed consent for participation was not required for this study in accordance with the national legislation and the institutional requirements.

## Author contributions

AY, JD, and SZ: conceptualization, methodology, formal analysis, investigation, and writing—original draft preparation. AY, JD, AD-B, and SZ: validation and writing—review and editing. JN and RA: resources. AY: data curation. AY, JD, JN, RA, and SZ: visualization. JD and SZ: supervision. JN, RA, and AD-B: project administration. All authors have read and agreed to the published version of the manuscript.
